# The APHIRM toolkit: an evidence-based system for workplace MSD risk management

**DOI:** 10.1186/s12891-019-2828-1

**Published:** 2019-10-30

**Authors:** Jodi Oakman, Wendy Macdonald

**Affiliations:** Centre for Ergonomics and Human Factors, School of Psychology and Public Health, La Trobe University, Bundoora, Australia

**Keywords:** Risk management, Musculoskeletal disorders, Psychosocial, Toolkit, Hazards

## Abstract

Musculoskeletal disorders (MSDs) continue as one of the largest occupational health and safety problems worldwide. One reason for this situation is that current workplace risk management practices fail to meet some important evidence-based requirements for effective reduction of MSD risk. In particular: they largely fail to address risk arising from psychosocial hazards; do not allow sufficient participation by workers; and often fail to control risk at its sources.

To address these deficiencies, A Participative Hazard Identification and Risk Management (APHIRM) toolkit has been formulated in accordance with both a framework developed by the World Health Organisation and implementation science principles. It comprises a set of online tools that include automated data analysis and reporting modules, and procedures to guide users through the five stages of the conventional risk management cycle. Importantly, it assesses both hazard and risk levels for groups of people doing a particular job, focusing on the job overall rather than only on tasks deemed to be hazardous. Its intended users are workplace managers and consultants responsible for occupational health and safety, with active participation from workers also. Resultant risk control interventions are customized to address the main physical and psychosocial hazards identified for the target job, and repetitions of the risk management cycle enables ongoing evaluation of outcomes in terms of both hazard and risk levels.

## Introduction

Musculoskeletal disorders (MSDs) are one of the largest Occupational Health and Safety (OHS) problems in many countries [1], including Australia where their annual total costs are calculated to be over $24 billion [2]. There is a general tendency for MSD incidence to increase as people age [3], but work-related hazard exposures significantly accelerate that underlying age-related increase [4]. In many countries, populations are ageing and workforce retirement ages are rising [5–7] so there is an increasingly urgent need for workplaces to implement evidence-based risk management practices that will reduce MSD risk more effectively [8].

One reason for this situation is that current workplace MSD risk management practices fail to meet some important evidence-based requirements for effective risk reduction [8–10]. To facilitate translation of research evidence into workplace practices, A Participative Hazard Identification and Risk Management (APHIRM) toolkit has been formulated [11–15]. The primary goal has been to develop a more effective set of workplace procedures for managing MSD risk, but the toolkit’s coverage of psychosocial hazards could be expected to benefit other stress-related outcomes also, including mental health [16, 17] and the quality of workers’ performance [18, 19].

### APHIRM toolkit design criteria

Research to develop this toolkit focused initially on addressing the following three evidence-to-practice gaps, all of which affect the efficacy of workplace risk management procedures and hence their *potential* effectiveness in reducing risk [20].

**Fig. 1:**
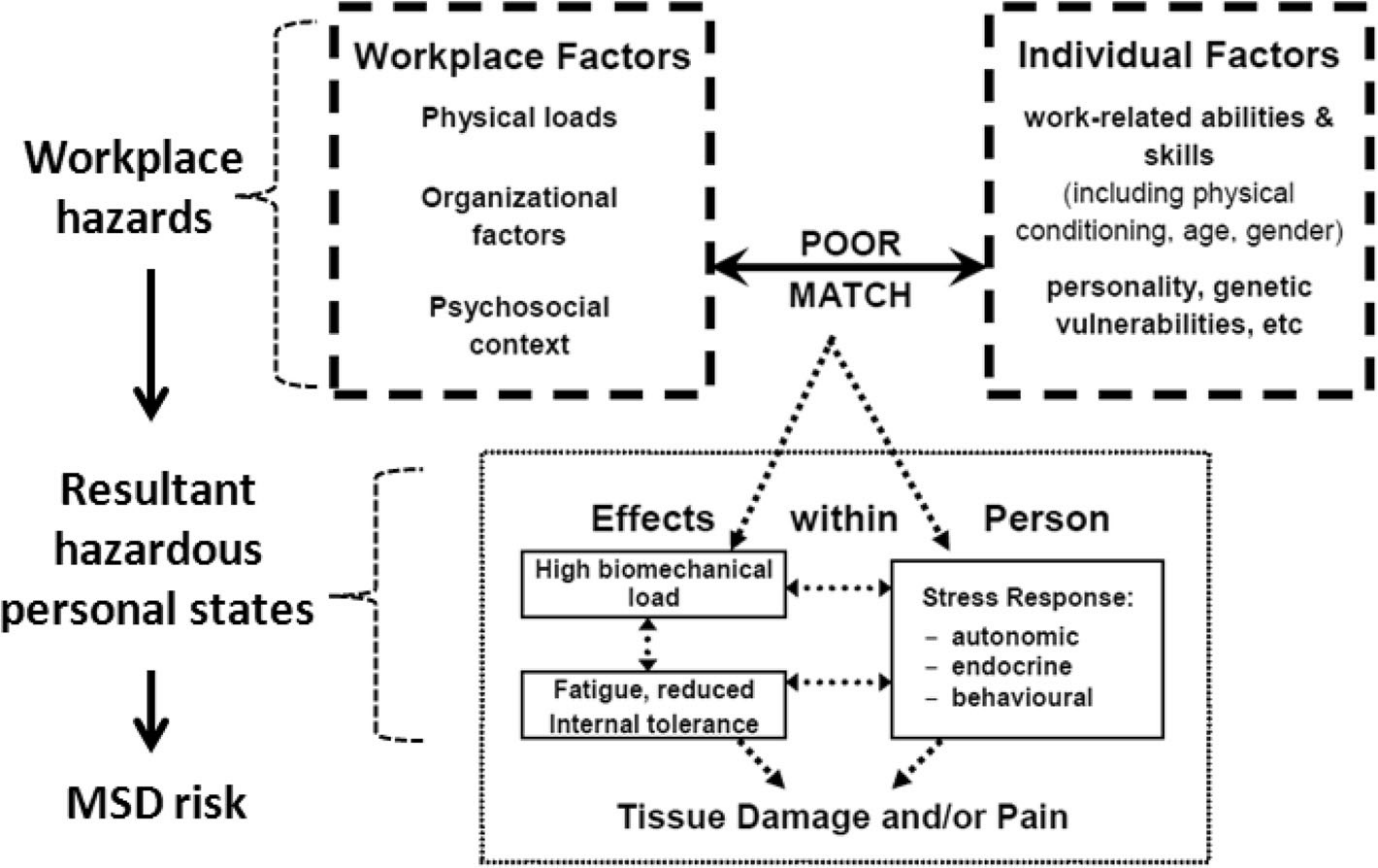
Factors affecting work-related MSD risk

Gap 1. Current workplace practices intended to reduce MSD risk typically focus narrowly on a few *physical* hazards such as heavy lifting and repetitive actions [8, 9, 11, 21–23]. Current practices generally fail to address MSD risk from work-related psychosocial hazards (also termed psychosocial ‘stressors’), which are characteristics of work organisation, job design and its social context that increase workers’ stress levels – e.g. excessive working hours, low autonomy and poor supervisor support. There is now a robust body of evidence that these psychosocial hazards can substantially increase MSD risk, and may interact with physical hazards [8, 11, 24–29] (see Fig. 1). Despite this evidence, MSD-related regulatory and guidance documents provide little information for workplace practitioners about how to manage MSD risk from psychosocial hazards [8, 11]. Also, the MSD risk management tools available for use by OHS professionals take little if any account of risk from psychosocial hazards, focusing primarily on physical hazards that arise from the performance of particular work tasks [8, 11, 30–33].

The APHIRM toolkit enables management of MSD risk from psychosocial hazards along with risk from the physical hazards that are the usual focus of MSD risk management. To achieve this, it assesses risk at the level of an overall job, rather than focusing just on specific tasks deemed to be hazardous. With psychosocial hazards this broader focus is essential because most of them are not task-specific; with physical hazards, the broader focus enables assessment of the cumulative effects of exposures across all tasks that form part of the job.

The toolkit’s broad coverage of a large and diverse set of potential hazards facilitates a more holistic approach to risk assessment, which has been identified as necessary for effective management of risks such as MSDs [8]. Focus on just one hazard at a time is appropriate when managing risks that arise mainly or entirely from a specific, observable source such as a hazardous substance, or a harmful form of energy such as electricity or loud noise. However, in cases such as MSDs (or mental disorders or major accidents) where risk results from the net effect of a large and variable set of hazards, some of which can interact with each other in affecting risk, effective management requires a broad, systems-based framework and more holistic assessment of risk from all relevant hazards *together* rather than in isolation from each other [8, 10, 12]. Following a similar rationale, many jurisdictions have now mandated a more holistic approach to safety management in industries where there is a risk of major accidents, rather than relying on the conventional OHS risk management paradigm [34, 35].

Gap 2. Workplace MSD risk management is more effective in reducing risk when workers are actively involved in the process [36–39]**.** In fact, assessment of risk from psychosocial hazards is not possible without worker participation; this is because many of these hazards are not observable by others, and also because risk is influenced by workers’ personal perceptions [16, 40]. Such evidence is not reflected in current workplace risk management practices, which rely largely on observation-based methods with minimal input from affected workers [8, 9, 11, 21]. Worker participation plays a key role in APHIRM toolkit procedures for both risk assessment and risk control.

Gap 3. Risk control actions are most effective when they address risk at its source, in accord with the conventional OHS hierarchy of risk control. However, current workplace practices focus largely on less effective MSD risk control strategies at the bottom of this hierarchy, such as training workers in ‘safe’ manual handling techniques [9] despite substantial evidence that this approach is ineffective in reducing risk [41–43]. Conventional formulations of the hierarchy are not easy to apply to MSD risk management [8, 44], so the APHIRM toolkit provides specific advice on risk control strategies for each of the various types of physical and psychosocial hazards, prioritising strategies that address MSD risk as close to its sources as possible.

The above toolkit design criteria are primarily concerned with efficacy. As such, they are not necessarily sufficient to achieve effectiveness in ‘real world’ conditions [20], and Takala (2018) [45] recently highlighted the need for greater attention to implementation-related issues if ergonomics interventions are to achieve their intended purpose. Effective workplace risk reduction requires that toolkit design should also take account of the characteristics of prospective users and the kinds of environments where implementation needs to occur, in accord with implementation science principles. For example, researchers in that domain have shown that effective interventions require clear processes and strong leadership, including support from senior management at all stages of the process [20, 46, 47]. The Quality Implementation (QI) framework shown in Table 1 summarises these principles; it was developed to support effective implementation of public health interventions [48]. These principles were used to guide toolkit design.

**Table 1:** APHIRM toolkit stages in relation to the WHO toolkits framework [48] and a Quality Implementation Framework [47]

APHIRM Toolkit Stages	World Health Organisation (WHO) framework and specifications for occupational health risk management toolkits	Quality Implementation (QI) Framework
**Stage 0.** **Getting started**	PLANNING AND IMPLEMENTATION: 1. Purpose of the toolkit 2. Defined target audience 3. Description of the working context … and content of the toolkit as outlined by the risk management cycle. … 4. How to get started: a) How to ensure management commitment; provide the rationale/business case b) Reinvigorating or setting up Labor/Management Committees and defining the role of safety committees in quick identification, communication and intervention …. in terms of structure and distribution of responsibilities TRAINING: 1. Models for training to cover requirements, recognition and continued good work practices. 2. Training modules on assessment, planning, implementation, evaluation and maintenance of prevention and control strategies. 3. Description of measures/indicators of success in implementing training. 4. Training recordkeeping, such as attendance records, course participation records, evaluation summaries	Phase One: Initial considerations regarding the host setting Assessment strategies *1. Conducting a needs and resources assessment* *2. Conducting a fit assessment* *3. Conducting a capacity/readiness assessment* Decisions about adaptation *4. Possibility for adaptation* Capacity-building strategies *5. Obtaining explicit buy-in from critical stakeholders and fostering a supportive community/organizational climate* *6. Building general/organizational capacity* *7. Staff recruitment/maintenance* *8. Effective pre-innovation staff training* Phase Two: Creating a structure for implementation *9. Creating implementation teams* *10. Developing an implementation plan*
**Stage 1.** **Identify main hazards and assess current risk**	**A. Identify potential workplace hazards** and/or high exposure work tasks or jobs and **assess the extent of risk** stemming from identified hazards, taking account of the severity of the hazard(s), and the duration of exposures to hazards and potential interactions between hazards.	Phase Three: Ongoing structure once implementation begins Ongoing implementation support strategies *11. Technical assistance/coaching/supervision*
**Stage 2.** **Identify local causes of main hazards**	B. For each significant hazard or combination of hazards: 1. identify possible means of eliminating the hazard(s) 2. where elimination is not possible, identify possible means of reducing the risk, often referred to as control options.	
**Stage 3** **Form action plan**	3. devise an action plan to reduce risks as much as possible, taking account of the following factors: a. for each particular hazard or group of interacting hazards, the level of risk presented (assessed at step 1 above) b. for each possible means of risk control (identified at step 2 above): • its probable effectiveness in eliminating or reducing risk • the practicability of implementing it • possible effects of its implementation on other hazards that lead to the same or different health outcomes 4. the perceptions and priorities of those who are at risk, concerning both the hazards that are most important to control and preferred means of managing them. 5. determine how it can best be evaluated.	
**Stage 4. Implement action plan**	C. Implement the action plan	
**Stage 5. Review – process evaluation**		*12. Process evaluation* *13. Supportive feedback mechanism*
**Stage 1 – REPEATED** (start of next risk management cycle)	D. Evaluate its impact E. Discuss the result of the evaluation and determine what can be learned from the successes and failures of the action plan. Revise and improve the action plan to target previously unaccounted risks and repeat the cycle.	Phase Four: Improving future applications *14. Learning from experience*

**Fig. 2:**
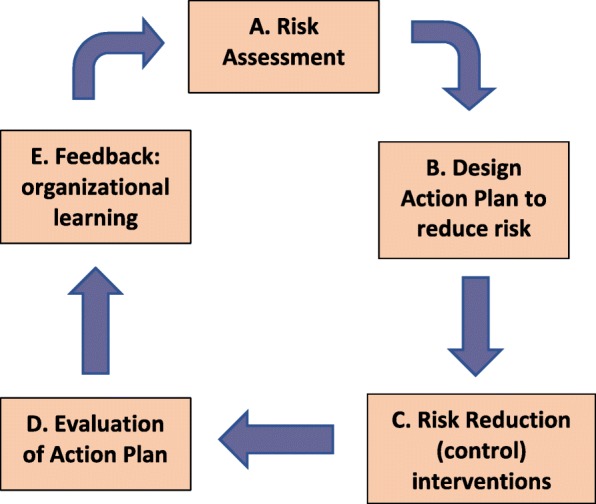
The WHO occupational health risk management framework for toolkits. (following: [49] p.14)

Further, toolkit procedures were formulated in accord with the framework and specifications developed by the World Health Organisation (WHO) for occupational health risk management toolkits. The WHO defines such a toolkit as “a solutions-oriented strategy applying practical tools for the control of a specific hazard or a specific risk”. A toolkit should comprise “a set of practical risk assessment procedures and related management guidance documents, including advice on simple risk control options” [49] pg. 13, and it should guide users through the steps of the conventional occupational risk management cycle as shown in Fig. 2 [49] (p.14).

Comparing the WHO toolkit and QI frameworks in Table 1, it can be seen that both of them incorporate some important implementation science principles. Their first parts are largely concerned with ensuring a good fit between the proposed intervention (in this case, the APHIRM toolkit) and what the QI framework terms the ‘host setting’. In the present case, two key aspects of the host setting are the intended users of the toolkit, and the workplace conditions in which it will be used. Importantly, both of these have been identified as the source of potential barriers to implementing more effective MSD risk implementation [8], as outlined below.

The toolkit’s intended users are workplace managers, including those with primary responsibility for OHS risk management. Unfortunately, there is a widespread assumption among managers that risk from psychosocial hazards is mainly relevant to mental rather than physical health; even among those in OHS specialist roles, many are unaware that psychosocial hazard exposures affect MSD risk [8, 50, 51]. This is problematic because awareness of the *need* for a particular change is an important pre-requisite for successful implementation of the change [52, 53]. In workplaces where musculoskeletal rather than mental health is recognized as the main OHS problem, managers are therefore unlikely to prioritise management of psychosocial hazards and stress. To overcome this barrier, the APHIRM toolkit integrates procedures to manage risk from psychosocial hazards with those targeting physical hazards, supported by information that explains the underpinning rationale. This approach was adopted despite the availability of *separate* guidance on workplace management of risk from psychosocial hazards and stress, because managers who did not see its relevance would be unlikely to implement it.

A second potential barrier to more effective MSD risk management arises from some characteristics of medium and large workplaces, which are likely ‘host settings’ for toolkit implementation. In such workplaces, direct responsibility for OHS risk management is usually delegated to someone designated as a specialist in this field, whose position in the organization is peripheral to the general management hierarchy. This is problematic because most of the physical and psychosocial hazards affecting MSD risk arise from characteristics of the job and the environment in which people work – that is, from factors that are the responsibility of general managers [8, 9, 50, 54]. A closely related problem is that MSD risk management procedures are typically not well integrated with more general management systems, which reduces their potential effectiveness [10, 55]. Addressing both these issues, the APHIRM toolkit specifies inclusion of a senior general manager in the MSDs risk management team, and risk control procedures actively involve general managers in development of action plans, thereby facilitating integration of MSD risk management with broader systems of management.

Table 1 presents the main stages of APHIRM toolkit procedures in relation to both the WHO toolkit and QI frameworks. The following section describes toolkit procedures within each of these main stages.

### Findings: outline of APHIRM toolkit procedures

#### Stage 0. Getting started

This preliminary stage is not part of the ongoing risk management cycle depicted in Fig. 2. Its primary purpose is to establish conditions that will be conducive to effective, sustainable implementation of the toolkit. As shown in Table 1, both the WHO and QI frameworks identify a range of factors that should be addressed at this stage, aiming to ensure that the intervention is appropriate for the host setting. Addressing this requirement, the ‘Getting Started’ stage of the toolkit includes components that briefly outline what its use entails, who its intended users are, how it supports compliance with legislation, and the expected benefits of using it.

This stage also includes procedures and resources that address point 4 in the WHO framework, and points 5 to 10 in the QI framework (see Table 1), aiming to ensure the support of senior management, to brief colleagues whose support will also be important, and to establish a small MSDs Risk Management Team (RMT) that will have direct responsibility for implementing toolkit procedures. Core RMT members should include a senior *general* manager as well as the person with primary responsibility for OHS management. Procedures are designed to manage MSD risk separately for different jobs, and for each job the RMT should recruit at least one worker representative, one manager, and where relevant a union representative also, to facilitate effective communications with all stakeholders.

Point 4 in the QI framework is about adaptability, which is inherent in various aspects of the toolkit. For example, its resources include a PowerPoint presentation for the RMT leader to customize by including current workplace data on MSD-related incidents and injuries and costs of MSD-related compensation claims, for use in demonstrating to top management the potential benefits of reducing MSD levels for selected jobs. Most importantly, all MSD risk control actions generated by toolkit procedures are customized to local requirements, based on extensive input from workers and their managers.

The QI framework also specifies that toolkits should include “Effective pre-innovation staff training” (point 7), and the WHO framework refers to a range of training modules covering all aspects of the risk management cycle. Design of the APHIRM toolkit and associated resources aimed to minimise training needs by ensuring that all required information is either directly available when needed, or easily accessible at that point. That is, it aimed to locate ‘knowledge in the world’ at points where it is required rather than relying mainly on ‘knowledge in the head’, since the latter depends heavily on user training and prior experience [56]. Nevertheless, initial training is provided and additional online training modules are under development.

#### Stage 1. Identify main hazards and assess overall risk level

At this first main stage of the continuing risk management cycle, existing hazard and risk levels are measured in accord with Step 1 of the WHO framework. This requires active participation from the workers involved, because levels of MSD hazard exposures are often difficult or impossible for others to observe accurately, particularly in the case of psychosocial hazards. The survey also documents musculoskeletal discomfort/pain levels, the importance of which is highlighted in the first of four formal position statements by the Scientific Committee on Musculoskeletal Disorders of the International Commission on Occupational Health: “Musculoskeletal discomfort that is at risk of worsening with work activities, and that affects work ability or quality of life, needs to be identified” (p.1) [57].

Workers in the target job therefore complete an anonymous survey. No individual data are visible to toolkit users, provided workgroup members respond to the survey online. (If paper surveys are used, the RMT will need to organise online data entry.) When limited literacy may present problems, it is recommended that survey questions are read aloud to groups of workers who each respond independently using separate input devices.

Workers rate their own exposures to a range of physical and psychosocial hazards (summarised in Table 2), and a mean rating is calculated for each hazard item. They also rate the frequency (out of 4) and severity (out of 3) of their physical discomfort/pain (last 6 months) in each of 5 body regions: neck/shoulders, arms, hands/fingers, middle/lower back, hips/bottom/legs/feet. A score out of 12 (4 × 3) for each body region is calculated, and these are summed to produce an overall score (out of 60) for each person reporting any discomfort/pain. These individual scores are used to generate a mean discomfort/pain score for the job, which serves as an indicator of current MSD risk, along with the percentage of workers who report some discomfort/pain. All calculations and reporting of results occurs automatically, driven by custom-designed software.

**Table 2:** Hazard items in the APHIRM toolkit survey

Physical task demands (12 items)	
Physical environment, equipment, OHS overall (6 items)	
Quantitative demands (3 items)	
Work pace (3 items)	
Emotional demands (2 items)	
Influence at work (1 item)	
Possibilities for development (4 items)	
Variation of work (1 item)	
Control over working time (1 item)	
Meaning of work (2 items)	
Predictability (1 item)	
Recognition (1 item)	
Role clarity (2 items)	
Role conflicts (2 items)	
Illegitimate tasks (1 item)	
Quality of leadership (2 items)	
Social support from supervisor (3 items)	
Social support from colleagues, Sense of community at work (2 items)	
Organisational justice (3 items)	
Job Satisfaction, Work-life balance (2 items)	

Grouping of psychosocial hazards in this table is based on Copenhagen Psychosocial Questionnaire (COPSOQ) categories. The 54 individual items are listed at www.aphirm.org.au; they are from various sources including but not confined to COPSOQ, as described in *APHIRM Toolkit Development Process*

To identify the main hazards, Spearman correlations between individual hazard ratings and individual discomfort/pain scores are calculated for each hazard item. Mean correlation values are ordered from highest to lowest, and those with the highest mean correlations are selected as ‘main’ hazards. This approach takes account of potential interactions between some hazards in their effects on risk. Also, any hazards with a mean rating of 4 or more (out of 5) are always included as ‘main’ hazards, regardless of their correlations with discomfort/pain. Correlations become unreliable with small data sets so when there are fewer than 15 individuals in the set, identification of main hazards is based only on hazard means. No more than 10 main hazards are reported, because it was found in pilot studies that dealing effectively with more than this in any one risk management cycle is usually impracticable due to limited availability of time and other resources.

The QI framework does not have a specific focus on risk management, but it refers to the need for “ongoing implementation support strategies” including “technical assistance/coaching/ supervision” (Point 11). The toolkit addresses this need by providing users with the option of coaching or supervision by La Trobe University researchers throughout the process of toolkit implementation.

### Stage 2. Identify causes and possible actions to reduce risk

This stage corresponds to Step 2 in the WHO toolkits framework. Having identified the main hazards for people doing a particular job, the next steps are to identify the local workplace factors that create or influence each of these hazards, and to propose possible changes that would eliminate or reduce risk from them. Again, workers’ participation plays an important role; they often have the most accurate and detailed information about local factors influencing these hazards, so are well placed to identify specific causes and suggest risk control actions.

All workers in the target job are invited to answer a series of online questions about the main hazards, and some of them are also invited to participate in a workshop to discuss these questions. An example of output from such a workshop is shown in Table 3, from a pilot study in the mining industry. This information is then used in Stage 3.

**Table 3:** Example of results from pilot implementation of APHIRM toolkit in a mining company: Stage 2 feedback from workgroup members

MAIN HAZARDS	CAUSAL FACTORS IDENTIFIED BY WORKGROUP MEMBERS	ACTIONS PROPOSED BY WORKGROUP MEMBERS
Often do very repetitive actions	• Constant use of the joystick • Twisting to see behind for reversing • Allocated to a dozer for the full roster cycle (7 days) • High turnover in crews (no relief opportunities) • Insufficient numbers of crew members to manage sickness, leave and breaks due to crew turnover • New starters engaged that don’t have appropriate skillset and experience	• Regular breaks out of the cab • Proactive task rotation (prior to onset of discomfort) to different jobs, for those workers who are interested • Training on other equipment to facilitate job rotation • Examine strategies to reduce turnover in the crew • Increase overall numbers in each crew • Engage the trainer/assessor in recruitment of new staff
Lack of opportunities for learning new skills and using existing skills	• Limited access to training opportunities • Workers with additional skills not being able to use these. • Not always clear about how work is allocated	• Training in use of other equipment for employees who want to work in other areas • People with skills to be able to rotate to other jobs • Transparent allocation of opportunities
Problems due to lack of promotion opportunities	• Difficult to become permanent • Tend to stay at the same level • No clear path to move to the next level • Only one trainer and assessor	• Development of individual plans for workers who want to move to higher levels • Investigate the role of performance reviews in this process • Develop and implement a clear and transparent process for workers who want to be trained on other equipment • Increase the number of trainers and assessors
Lack of feedback on performance	• Workers feel they are doing a difficult job, but this is not always recognised as valuable, some jobs considered more important	• Workers reported that this has improved but asserted that feedback needs to be meaningful
Opinions differ on ‘correct’ way to do some tasks	• Differences in the way things are done between crews • Rework required because of inconsistent practices between the different crews	• Implementation of “Dozer Playbook” reported as an action to address this issue. This process was designed to ensure more early reporting from workers was positive
Often hold or grip things with hands or fingers	• Inherent part of the job, concerned with operating the controls and also for bracing to reduce load on back	• No ideal solutions identified but rotation of tasks would change the exposure to this hazard • Improved blasting to reduce the exposure to jolts and jars due to working in hard material
Senior management attitudes	• Workers feel they are not respected and do not have a voice • Senior managers not visible • New dozers reported to be coming but still have not arrived after 12/18 months • Projects can take a long time to be implemented	• Greater visibility of senior managers so that they understand the issues faced by workers in their work when decisions are made that impact how they do their job • Communication from senior managers, even when things could be changed, workers want to know good and bad news and an explanation underpinning decisions • Provide regular updates to workers on projects, even when projects are slow/delayed • Have input to new equipment prior to being ordered
Work stations and workspace	• Overcrowding in crib hut	• Bigger crib hut to be provided

### Stage 3. Formulate an action plan

Using a pre-populated template generated by toolkit software, the RMT leader completes a summary of workers’ suggestions about how to eliminate or reduce risk from each of the main hazards. The full RMT then meets to review these suggestions and formulate an action plan. To help them in this task, the toolkit suggests risk control actions specific to each of the main hazards, recommending actions that will address risk at its source in accord with principles underpinning the hierarchy of risk control.

### Stage 4. Review and implement action plan

RMT leaders brief general managers, including those responsible for financial resources, on the proposed action plan and its rationale. Managers then review and prioritise the proposed actions, with reference to toolkit guidance on hierarchy of risk control principles and to the legal requirement to do everything ‘reasonably practicable’ to eliminate or reduce risks to workers’ health and safety [58]. They need to identify the people responsible for required actions and to establish a realistic timeframe, bearing in mind local contextual factors. In doing this, they are encouraged to consider how best to integrate risk control actions with existing business practices and management procedures, in order to promote more effective and sustainable risk management [55, 59].

Throughout this stage the toolkit facilitates good communications with workers in the target job because, having involved them in the hazard and risk assessment, it is important to maintain morale by keeping them informed about the action plan and progress implementing it. Also, workers and their line managers are invited to provide feedback so that any problems inadvertently created by risk control actions can be dealt with promptly, and to support process evaluation of toolkit procedures more generally.

### Stage 5. Review and evaluation

This last stage of the risk management cycle (Step 5 in the WHO framework) overlaps Stage 1 of the following cycle. Team leaders are guided by toolkit protocols through a review of how well procedures were implemented, based on feedback from Stage 4 as well as their own observations. On this basis, they identify any problems and possible means of avoiding these in future, corresponding with points 12–14 in the QI framework.

Two sets of information are used in evaluating outcomes:

1. satisfaction ratings of workgroup members and their managers (from Stage 4 feedback);
2. most importantly, MSD hazard and risk profiles measured during Stage 1 of the following risk management cycle.

It is expected that this cycle will be repeated annually.

### APHIRM toolkit development process

The toolkit has been developed through research in a range of industry sectors where MSD claim rates are among Australia’s highest. Initial focus was on development and validation of the toolkit’s hazard and risk assessment component (Stage 1). The risk assessment methods used initially included: physical measurements and video-based analyses by an experienced ergonomist; observation-based ratings of physical task demands by non-experts; and a survey of employees that included standard measures of both stress and fatigue as well as ratings of discomfort/pain and hazard exposures.

Based on reviews of research literature, survey items were developed to cover physical hazards arising from manual handling tasks, and items were developed or selected from existing surveys to cover psychosocial hazards. All of the Work Organisation Assessment Questionnaire (WOAQ) was used, since this was developed specifically for use in manufacturing industry where MSD risk is relatively high (Griffiths et al., 2006 [60]). Additional items were included to improve coverage of workload, factors affecting work rates, co-worker relationships, and perceived influence or control levels, drawing from various sources including the Job Content Questionnaire (Karasek et al., 1998 [61]), the Job Diagnostic Survey (Hackman & Oldham, 1974 [62]), a Work Characteristics survey (Marlow et al., 2005 [63]), and our previous research on effects of highly repetitive, externally-paced work on employee wellbeing [64, 65].

Survey responses were obtained from employees in four large workplaces in two industry sectors where MSD risk is high (total *n* = 493), and factor analysis identified constructs for use in multivariate regression analyses to identify the subsets of hazard items most strongly predictive of discomfort/pain scores. The resultant highly significant regression model included scores for: physical task demands; overall WOAQ score; working faster to meet deadlines or targets; and quantitative workload. Scores on these constructs reflect hazard exposures across the *whole job*, and these were much stronger predictors of discomfort/pain than were scores from the risk assessment methods used to assess the physical demands of *particular tasks*. Also, high correlations were found between discomfort/pain scores for each of five body regions and numbers of workplace MSD incident reports for each of these five regions, indicating that discomfort/pain score is a valid index of MSD risk. Details of this research have been reported [14], and a summary is in preparation for publication.

Following initial development of the survey as outlined above, drafts of the APHIRM Toolkit Stages 1 to 3 were pilot tested in a variety of workplaces in other industry sectors with high MSD risk (aged care, mining, local government). During this second stage of toolkit development the survey was amended by addition of items from the Copenhagen Psychosocial Scale (COPSOQ), expanding its coverage of psychosocial hazards to accommodate the wider range of job types. Also, wording of some items was amended to clarify intended meanings. These amendments were essential because average literacy levels were not high in the kinds of jobs being targeted, and because it became evident that some questions were likely to be understood differently by people in different jobs (consistent with results of Clausen et al., 2019 [66]). Table 2 outlines content of the current APHIRM Toolkit survey, with items grouped in accord with COPSOQ categories. Note, however, that workplace risk management is based on scores for individual survey items rather than general constructs, since the greater detail in individual items can more easily be linked to specific workplace sources of risk at Stage 2 of the risk management cycle.

During piloting of Stages 1 to 3 of the toolkit, a range of barriers to effective implementation were encountered. In particular, lack of top management commitment was identified as a potential barrier; this is essential, particularly to ensure that workers are given the 10 min required to complete the survey, and to support implementation of risk control actions. A second major barrier was the intimidating nature of the toolkit itself, which initially resembled a paper-based user manual, densely packed with information. Third, use of paper-based surveys requires someone to enter all responses into an Excel spreadsheet prior to analysis and reporting – initially by bespoke software using MS Excel macros. Although the resultant report was greatly appreciated, the data entry task was both time-consuming and error prone, and often seen as impractical in view of very limited staff resources.

To investigate barriers to implementation in prospective ‘host settings’ more systematically, a project was undertaken in industry sectors where MSD risk is high, aiming to review the main “initial considerations regarding the host setting” in accord with the QI framework (Table 1, points 1 to 3). This entailed semi-structured interviews with 67 people representing a range of management levels in 19 large organisations. Most of the interview questions were about general MSD risk management issues. It was found that: risk from work-related psychosocial hazards was not being adequately assessed or controlled; the most commonly reported risk control actions focused on changing workers’ behaviours rather than addressing risk from work-related hazards at their sources in accord with the hierarchy of risk control; and existing workplace risk management procedures were highly fragmented. This latter situation is problematic for health disorders such as MSDs that are affected by a diverse and potentially interacting set of hazards, because assessing the severity of any individual hazard in isolation from the others is not necessarily a good indicator of MSD risk [50].

In addition, near the end of each of the 67 interviews the participant was presented with a YouTube presentation that briefly explained the nature and role of psychosocial hazards and associated risk management issues, followed by a brief account of the rationale, structure and main components of the APHIRM toolkit. The aim was to enable participants to understand the toolkit rationale sufficiently to assess its general strengths and to suggest likely barriers to implementing it in workplaces such as theirs. The factors they perceived as barriers to implementation confirmed previous findings, focusing on difficulties obtaining senior management commitment, and limited resources both financially and in staff skill levels. Importantly, factors they perceived as strengths included: covering risk management of mental as well as musculoskeletal disorders; involving workers in risk management; and complementing existing OHS management systems.

Based on all the above evidence, it was concluded that a key requirement for effective implementation is that the toolkit be presented in an easily usable format and sources of help for users are easily available. To achieve this, we have transformed the toolkit into a web-based format for online use, applying fundamental principles of human-computer interface design [67, 68] in collaboration with designers who are technical experts in the development of large, complex websites.

## Discussion: current status of the APHIRM toolkit

The current, web-based toolkit has greatly simplified users’ interactions with the toolkit; all procedures are now managed online, with the user supported by specific prompts, action checklists and feedback. It has removed the need for manual data entry by enabling online survey completion via a mobile phone or other such device; workers and their managers can also provide feedback online. Data analysis and reporting is still performed automatically, now using online software. Many components of the toolkit can be printed if required, including the survey so that paper-based survey completion is still possible if preferred (although this would require subsequent manual data entry). An APHIRM toolkit website (www.aphirm.org.au) has been established to provide access to the toolkit and to online resources for workplace practitioners. It is now available for trialing on condition that users complete an initial one-day training course, and that resultant anonymous data are available to La Trobe University researchers for use in ongoing research.

## Summary and conclusions

If the current incidence of work-related MSDs is to be reduced significantly, workplace practices will need to undergo substantial change. An extensive body of peer-reviewed research has identified a large and diverse range of work-related hazards affecting MSD risk, and requirements for effective workplace interventions. However, current workplace practices do not reflect this evidence. More effective risk management requires: expansion of coverage beyond the physical hazards of manual handling tasks to encompass psychosocial hazards of the overall job; a higher level of worker participation in both risk assessment and risk control procedures; and giving higher priority to actions that control risk at its sources. Also, MSD risk management is more likely to be effective when it is better integrated with general management systems rather than being a peripheral activity.

The APHIRM toolkit addresses the need for such changes. Its structure accords with a framework specified by the WHO, and its development drew on evidence from extensive workplace trials as well as implementation science principles that promote sustainable uptake in ‘real world’ environments. It provides a set of online tools, including automated data analysis and reporting modules and guidance to support workplace managers and OHS practitioners at all stages of the standard risk management cycle. It also enables workers to be active participants in assessing risk and identifying risk control actions customized to the hazard profiles of their job. Repeated application of the toolkit risk management cycle enables ongoing evaluation of outcomes in terms of both MSD risk and hazard levels, facilitating continual improvement. Overall, the APHIRM toolkit appears to be unique in the comprehensiveness of its approach to workplace management of MSD risk.
